# SOCS1, a Negative Regulator of Cytokine Signals and TLR Responses, in Human Liver Diseases

**DOI:** 10.1155/2010/470468

**Published:** 2010-09-02

**Authors:** Minoru Fujimoto, Tetsuji Naka

**Affiliations:** Laboratory for Immune Signal, National Institute of Biomedical Innovation, 7-6-8 Saitoasagi, Ibaraki, Osaka 567-0085, Japan

## Abstract

Toll-like receptor (TLR) signaling pathways are strictly coordinated by several mechanisms to regulate adequate innate immune responses. Recent lines of evidence indicate that the suppressor of cytokine signaling (SOCS) family proteins, originally identified as negative-feedback regulators in cytokine signaling, are involved in the regulation of TLR-mediated immune responses. SOCS1, a member of SOCS family, is strongly induced upon TLR stimulation. Cells lacking SOCS1 are hyperresponsive to TLR stimulation. Thus, SOCS1 is an important regulator for both cytokine and TLR-induced responses. As an immune organ, the liver contains various types of immune cells such as T cells, NK cells, NKT cells, and Kupffer cells and is continuously challenged with gut-derived bacterial and dietary antigens. SOCS1 may be implicated in pathophysiology of the liver. The studies using SOCS1-deficient mice revealed that endogenous SOCS1 is critical for the prevention of liver diseases such as hepatitis, cirrhosis, and cancers. Recent studies on humans suggest that SOCS1 is involved in the development of various liver disorders in humans. Thus, SOCS1 and other SOCS proteins are potential targets for the therapy of human liver diseases.

## 1. Introduction

Proper and coordinated activation of immune signal pathways is required for immune responses, including eradication of invading pathogens. Toll-like receptor (TLR)- and cytokine receptor-mediated signaling are involved in innate and subsequent adoptive immunity. Aberrant and/or sustained activation of immune signal pathways may result in serious disorders such as septic shock, autoimmunity, and cancer. Thus, immune signals must be tightly regulated for preventing overactivated immune responses. A number of regulatory mechanisms on immune signaling pathways have been reported. A family named suppressor of cytokine signaling (SOCS) represents a negative regulator for various cytokine signaling (Table 1) [1]. SOCS proteins play important roles in maintaining organ homeostasis by preventing the harmful cytokine responses in various organs [2]. In this paper, we will focus on SOCS1, a member of SOCS family, which plays a key role in the negative regulation of both cytokine receptor- and TLR-mediated signaling. We will further discuss the importance of SOCS1 in the pathogenesis of liver diseases.

## 2. Regulation of Immune Signal Pathways by Suppressor of Cytokine Signaling (SOCS)

### 2.1. Inhibition of Cytokine Signaling by SOCS

Cytokine receptor-mediated signaling critically regulates cellular functions including proliferation, differentiation, and survival. SOCS proteins are originally discovered as cytokine-induced proteins that negatively regulate cytokine receptor signaling (Table 1) [1]. This regulation by SOCS proteins prevents the harmful overactions mediated by cytokine signaling. The physiological roles of SOCS proteins have been extensively investigated by the studies using knockout animals [1, 2]. 

**Figure 1:**
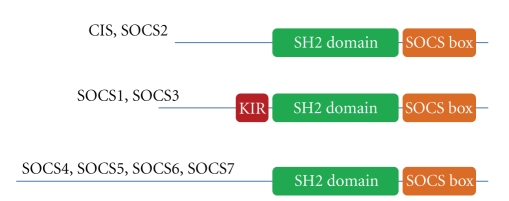
Schematic structure of SOCS proteins. SOCS proteins are structurally characterized by a central SH2 domain, a docking motif to tyrosine-phosphorylated proteins, and a c-terminal SOCS box that recruits Elongin B/C complex. SOCS1 and SOCS3 also possess a KIR domain, which plays an important role in inhibition of JAK kinase activity. The length of N-terminal domain varies between SOCS proteins and SOCS4-7 possesses relatively long N-terminal domain.

In mammals, eight members of SOCS proteins (SOCS1 to SOCS7 and CIS) have been reported [1]. These proteins consist of two conserved motifs, a central SH2 domain and a C-terminal SOCS box (Figure 1) [1]. SOCS1 and SOCS3 possess a kinase-inhibitory region (KIR) domain that is critical for inhibition of kinase activity [1]. The SH2 domain of SOCS proteins is a crucial component for association of SOCS proteins with phosphorylated tyrosine residues on tyrosine kinases or cytokine receptors [1]. This association inhibits cytokine signaling by suppressing kinase activity or by masking docking sites for adaptor molecules on the receptors. In addition, the SOCS box recruits a complex containing elongin B, elongin C, cullin-5, RING-box-2, and E2 ligase via its subdomains, a B/C box and a Cull5 box, and mediates ubiquitination of SOCS-bound proteins for proteasomal degradation [5]. 

Among SOCS proteins, SOCS1 is a prototype molecule. A number of studies indicate that SOCS1 preferentially binds to JAK kinases via its SH2 domain and inhibits signaling by shutting down JAK kinase activity via the KIR domain as well as by promoting JAK degradation via the SOCS box [2]. Given the role of JAK kinases in signaling of cytokine receptors, mice lacking SOCS1 exhibit hypersensitivity to a variety of cytokines, including IFN-*α*, IFN-*γ*, IL-2, IL-4, IL-7, IL-12, and IL-15 (Table 1) [1, 2]. SOCS1 deficient mice are born normally, but die within three weeks of age due to lymphocyte-dependent multiorgan inflammatory disease [1, 2].

### 2.2. SOCS1 Negatively Regulates TLR Signaling

The expression of SOCS1 is induced by various cytokines, including IL-4 and IFN-*γ*, and also by TLR ligands, such as LPS and CpG-DNA (Table 1) [3]. TLR ligands induce SOCS1 expression directly through the activation of early growth response-1 (Egr-1) [6] and/or indirectly through cytokines, including IL-6 and IFN-*β* induced by initial TLR signaling [3]. This finding raises the possibility that SOCS1 regulates TLR signaling. In accordance with this, enforced expression of SOCS1 results in reduced response of cells to TLR ligands. More importantly, SOCS1 deficient mice are hypersensitive not only to cytokines but also to TLR ligands [7, 8]. Upon stimulation with LPS, a TLR4 ligand, or CpG-DNA, a TLR9 ligand, SOCS1 deficient macrophages produce an increased amount of inflammatory cytokines, including TNF-*α*, IL-6, and IL-12. Furthermore, LPS tolerance, refractoriness to second challenge with LPS after initial LPS exposure, is not induced in SOCS1 deficient mice. Although IFN-*γ*/STAT1 pathway is the major target for SOCS1, SOCS1 deficient cells lacking IFN-*γ* or STAT1 still exhibited enhanced response to LPS [7, 8]. This suggests that the hypersensitivity of SOCS1 deficient cells to LPS is largely due to their dysregulated response to TLR signaling, but not IFN signaling secondarily induced by TLR signaling. Thus, SOCS1 directly induces the negative regulation of TLR signaling [3, 5].

## 3. Mechanisms of Negative Regulation of TLR Signaling by SOCS1

**Figure 2:**
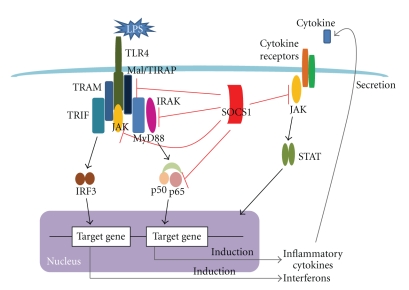
Proposed inhibitory mechanisms of SOCS1 on TLR signaling. The absence of SOCS1 results in heightened response to TLR ligands. Possible mechanisms of action of SOCS1 proposed so far are outlined here. Upon induction, SOCS1 binds to Mal/TIRAP and mediates its degradation via proteasomal pathway (1). SOCS1 binds to IRAK and may modulate its activity (2). SOCS1 binds to p65 subunit of NF*κ*B and targets it for proteasomal degradation (3). SOCS1 inhibits JAK2 activated directly after TLR stimulation (4). SOCS1 regulates TLR-mediated response indirectly by inhibition of TLR-induced cytokines such as IFN-*β* (5).

SOCS1-mediated negative regulation of TLR signaling has been reported by a number of studies [3]. Although the major mechanism by which SOCS1 regulates TLR-mediated responses remains a matter of debate, several mechanisms have been proposed as outlined below (Figure 2). 

### 3.1. Regulation of TLR-Mediated Cytokine Signaling by SOCS1

The activation of TLR signaling results in the induction of inflammatory cytokines such as TNF-*α*, IL-6, and IFN-*α*/*β*. Given the role of SOCS1 in inhibition of cytokine-mediated signaling, one of the most representative functions of SOCS1 is to regulate intracellular signaling activated by cytokines which are induced by initial TLR signaling. Indeed, several reports demonstrated that the signaling of IFN-*β* induced by the first TLR stimulation is the critical target of SOCS1 [9–11]. In accordance with this, SOCS1 deficient cells are highly sensitive to type I IFNs, and lethal disease in SOCS1 deficient mice is partly mediated by the dysregulation of IFN-*α*/*β* signaling [12].

### 3.2. Association of IRAK with SOCS1

Two independent studies have shown that LPS-induced proinflammatory cytokine production and NF-*κ*B activation are inhibited in cells overexpressing SOCS1 [7, 8]. Among signaling molecules that bridge between TLR and NF-*κ*B, IL-1 receptor-associated kinase 1 (IRAK1) is a target of SOCS1. SOCS1 directly associates with IRAK1 via its SH2 domain [7].

### 3.3. Regulation of Mal/TIRAP Protein Expression by SOCS1

The TLR adaptor molecule Mal/TIRAP is also a target of SOCS1 [13]. TLR stimulation induces Btk-dependent tyrosine-phosphorylation of Mal and generates a binding site for SH2 domain of SOCS1. Subsequent interaction between SOCS1 and Mal induces ubiquitination and proteasomal degradation of Mal, resulting in abrogation of TLR/Mal-dependent NF-*κ*B activation [13]. A recent report has shown that SOCS1 also regulates the intracellular protein levels of Mal in hepatocytes, which inhibit intracellular uptake of LPS by hepatocytes [14].

### 3.4. Regulation of NF-*κ*B Activation by SOCS1

SOCS1 also regulates TLR-induced NF-*κ*B activation by direct interaction with p65 subunit of NF-*κ*B upon stimulation with LPS. SOCS1 downregulates p65 protein levels by ubiquitin-mediated degradation of p65 [15].

### 3.5. Regulation of TLR-Induced JAK2 Activation by SOCS1

Several reports demonstrated that JAK2 in macrophages contributes to the induction of proinflammatory cytokines by LPS stimulation [16, 17]. Since JAK2 is a major target of SOCS1, it is likely that the inhibitory effect of SOCS1 on LPS signaling is partially due to the binding of SOCS1 to JAK2 in TLR4/JAK2 pathway [17].

## 4. SOCS1 in Liver Pathophysiology

SOCS1 expression is induced in hepatocytes by a variety of exogenous stimulation such as IL-6 and IFN-*γ* during liver pathobiology. SOCS1 regulates various intracellular signaling pathways in hepatocytes, which may modulate liver pathophysiology (summarized in Table 2). 

### 4.1. Protective Roles of SOCS1 in Hepatitis

SOCS1 deficient mice display a fatal neonatal disease, which is characterized by aberrant activation of T cells and multiple organ injury. Hepatic inflammation accompanied by fatty degeneration and hepatocyte necrosis is the major cause of the death in SOCS1 deficient mice. This fulminant hepatitis in SOCS1 deficient mice is due to exacerbated activation of hepatic lymphocytes, including NKT cells [18], and increased sensitivity of hepatocytes to inflammatory cytokines such as IFN-*γ* [19]. 

SOCS1 expression is also important for suppression of hepatitis in adult mice. Concanavalin A- (ConA-) induced hepatitis is a murine model of T cell-mediated acute hepatitis. In this model, IFN-*γ*/STAT1 pathway accelerates liver injury, while IL-6/STAT3 ameliorates disease [20]. Both SOCS1 and SOCS3 are induced in the liver after ConA injection [20] and provide reciprocal regulation in IFN-*γ*/STAT1 and IL-6/STAT3 pathway [21]. Compared to wild type mice, mice with specific deletion of SOCS1 in hepatocytes (hepatocyte-specific SOCS1 conditional KO mice) exhibit severe ConA-induced hepatitis with increased mortality [22]. Conversely, SOCS3 conditional KO mice exhibit reduced liver injury [23]. Collectively, SOCS1 expression in the liver prevents fatal hepatitis via the suppression of exacerbated liver inflammation.

### 4.2. Protective Roles of SOCS1 in Liver Fibrosis and Carcinoma

Dimethylnitrosamin (DMN) treatment induces liver fibrosis in mice. SOCS1 heterozygous mice exhibit enhanced liver damage, severe liver fibrosis, and increased mortality after the treatment with DMN, compared to wild type mice. After treatment with diethylnitrosamin (DEN), a chemical agent that induces hepatocellular carcinoma (HCC), SOCS1 heterozygous mice developed more tumors than wild type mice [24]. These findings suggest that endogenous SOCS1 prevents liver fibrosis and hepatocarcinogenesis.

### 4.3. SOCS Proteins Regulate Liver Regeneration

MyD88-dependent innate immune signaling induces inflammatory cytokines, including TNF-*α* and IL-6, to initiate liver regeneration after partial hepatectomy (PH) [25, 26]. SOCS genes are induced through MyD88 during liver regeneration [26, 27]. Produced IL-6 during liver regeneration activates STAT3, which is an important component to induce hepatocyte proliferation as well as a crucial target of SOCS1 and SOCS3 in hepatocytes. Enforced SOCS1 or SOCS3 expression in hepatocytes inhibits STAT3 activation and hepatocyte proliferation induced by IL-6, hepatocyte growth factor (HGF), and epidermal growth factor (EGF), which results in the suppression of liver regeneration [28, 29]. Mice lacking SOCS3 in hepatocytes exhibit enhanced STAT3 activation, hepatocyte proliferation, and liver weight restoration after PH [30]. Thus, SOCS molecules negatively regulate physiological proliferation of hepatocytes after PH.

### 4.4. Role of SOCS Proteins in Metabolic Syndrome

Proinflammatory cytokines are key factors to develop metabolic syndrome, including insulin resistance. Several lines of evidence suggest that inflammatory cytokines exacerbate insulin resistance via SOCS protein induction in the liver [31]. The expression of SOCS1 and SOCS3 is increased in a murine model of obesity [32]. *In vitro* experiments using cells overexpressing SOCS suggest that members of the SOCS family, such as SOCS1, SOCS3, and SOCS6, inhibit insulin signaling directly by interacting with insulin receptor and/or IRS [31]. *In vivo*, Adenoviral overexpression of SOCS1 or SOCS3 in the liver enhances insulin resistance and fatty acid synthesis [32]. In addition, cells lacking SOCS1, SOCS3, or SOCS7 exhibit increased sensitivity to insulin [33–36]. These results suggest that the induction of SOCS1 and other SOCS proteins in hepatocytes plays an important role in hepatic metabolism and pathogenesis of metabolic syndrome.

## 5. SOCS1 in Human Liver Disease

Studies using animal models suggest that inadequate induction or impaired expression of SOCS1 may induce liver diseases in humans. Thus far, although inactivating mutations in SOCS1 gene were detected in a subset of lymphoma cells [2], such mutations have not been observed in hepatic disorders. Nevertheless, previous studies have shown that SOCS1 CpG islands in human primary hepatocellular carcinomas (HCCs) are frequently methylated, suggesting that the epigenetic silencing of SOCS1 participates in tumor growth of HCCs [37, 38]. Interestingly, SOCS1 methylation is highly prevalent in HCC with HCV infection but not with HBV infection [39]. In addition, SOCS1 gene methylation is also detected in patients with hepatitis C [24]. These results suggest that endogenous SOCS1 is a tumor suppressor of HCCs and epigenetic modification of SOCS1 expression by HCV infection leads to the progression of hepatic inflammation and the progression of HCCs. 

On the other hand, genetic polymorphism on SOCS1 promoter region may impair SOCS1 induction. Indeed, a recent study demonstrated that the SOCS1 polymorphisms in Caucasians are associated with alteration in body mass index [40]. Although it remains unclear whether SOCS1 polymorphisms modify SOCS1 expression in the liver and SOCS1 polymorphisms influence insulin sensitivity and lipid metabolism, SOCS1 polymorphisms might be associated with metabolic syndrome in humans.

## 6. SOCS3 in TLR Signaling and Human Liver Diseases

Overexpression studies have shown that both SOCS1 and SOCS3 exhibit similar inhibitory functions on various cytokine signaling *in vitro* (Table 1). However, SOCS1 and SOCS3 are not functionally interchangeable. Interestingly, SOCS3 deficient mice are embryonic lethal, due to placental insufficiency as a result of hypersensitivity to LIF [1]. Additional studies using SOCS3 conditional KO mice revealed that SOCS3 is required for negative regulation of IL-6, leptin, and G-CSF signaling [3]. These results indicate that SOCS3 has nonredundant physiological inhibitory functions in cytokine signaling, including IL-6, LIF, leptin, and G-CSF signaling. 

Several reports have shown that SOCS3 regulates TLR signaling. LPS treatment strongly induces SOCS3 expression in macrophages and hepatocytes [41]. In addition, like SOCS1, SOCS3 inhibits TLR-mediated responses [2]. However, the action of SOCS3 in TLR signaling may be complex, because there are conflicting reports describing that SOCS3 has little or even enhancing effect on TLR response [11, 42]. Moreover, mice lacking SOCS3 in myeloid cells are resistant to LPS-induced lethal shock [22]. Nevertheless, it is of interest to elucidate the precise function of SOCS3 in TLR response, since accumulating evidence suggests that SOCS3 is important for the pathology of hepatic diseases.

As described earlier, SOCS3 is induced in the liver during liver regeneration. Hepatocyte proliferation and hepatic weight restoration are enhanced in hepatocyte-specific SOCS3 knockout mice [30]. ConA or DMN treatment induces excessive fibrosis in hepatocyte-specific SOCS3 knockout mice, although hepatic inflammation is ameliorated in these mice [43]. In addition, hepatocyte-specific SOCS3 knockout mice develop more liver tumors than wild type mice after DEN treatment [23, 30]. Stimulation of cytokines such as IL-6 induces constitutive activation of STAT3 in SOCS3 deficient hepatocytes. Given the crucial role of IL-6/STAT3 pathway in HCC development [44, 45], this might be the mechanism of enhanced tumorigenesis in hepatocyte-specific SOCS3 knockout mice. These findings suggest that SOCS3 in hepatocytes regulates hepatocyte homeostasis, including survival and proliferation, which further contributes to the regulation of fibrosis and carcinogenesis in the liver. Notably, SOCS3 in hepatic T cells may have a different role in the regulation of hepatic inflammation. Conditional deletion of SOCS3 in T and NKT cells exacerbates ConA-induced hepatitis [46], while transgenic overexpression of SOCS3 in T and NKT cells [46, 47] or intraperitoneal administration of cell-penetrating SOCS3 in mice [48] prevents ConA-induced liver injury. 

In humans, SOCS3 is implicated in HBV- and HCV-induced pathology of the liver. In contrast to the observation in SOCS1, SOCS3 expression is upregulated in the livers of HBV [49] and HCV patients [23, 50]. HBV X protein enhances SOCS3 expression in hepatocytes [51]. HCV core protein induces SOCS3 expression in hepatoma cells [52, 53], while it inhibits SOCS1 expression [54]. Enhanced SOCS3 expression may prevent HCC development, but may induce insulin resistance [53], immune dysfunction [49], and refractoriness to IFN therapy [52]. Interestingly, in the liver of HCV patients with HCC, SOCS3 expression is increased in noncancerous region but remained low in HCC region [23, 30]. This observation suggests that SOCS3 in hepatocytes is somehow silenced during tumorigenesis in HCV patients. Thus, while further studies are required, SOCS3 could be an attractive target for the therapy of human liver diseases.

## 7. Conclusion

Although the mechanism by which SOCS1 inhibits TLR signaling is not fully understood, accumulating evidence indicates that the absence of SOCS1 strongly enhances TLR responses. Since SOCS1 is induced in the liver by multiple factors including cytokines, TLR ligands, and insulin, SOCS1 should regulate both cytokine and TLR signaling under various physiological and/or pathological conditions. In accordance with this, mice lacking SOCS1 are sensitive to a variety of liver diseases including hepatitis, liver cirrhosis, and HCC. Moreover, silenced SOCS1 expression due to CpG methylation is involved in pathogenesis of hepatitis C, HCC, and many other types of human cancers [2]. It is also possible that impaired SOCS1 expression may underlie the pathogenesis of other human liver diseases induced by infection, autoimmunity, drugs, and alcohol. In contrast, it has been shown that an excessive induction of SOCS1 by TLR ligands and cytokines contributes to the alteration of insulin sensitivity in metabolic syndrome. Future studies on SOCS1 and other SOCS proteins will provide insight into the pathobiology of human liver diseases and develop new strategies for the treatment of acute and chronic liver diseases. 

## Figures and Tables

**Table 1:** Inducing factors of SOCS family proteins and suppressed signaling by SOCS family proteins (see [1, 3, 4]).

SOCS family	Inducers	Suppressed Signaling*
CIS	IL-2, IL-3, EPO, GM-CSF, GH, Prolactin	IL-2, IL-3, EPO, GH, Prolactin

SOCS-1	IL-2, IL-3, IL-4, IL-6, IL-7, IL-9, IL-10, IL-13, IL-15, IL-21, IFN-*α*/*β*, IFN-*γ*, LIF, TNF-*α*, TGF-*β*, EPO, G-CSF, SCF, GH, TSH, Prolactin, CNTF, Cardiotrophin, Insulin, LPS, CpG DNA	**IL-2**,** IL-4**, IL-6,** IL-7, IL-12, IL-15**, **IL-21**, **IFN-** **α**/**β**, **IFN-** **γ**, LIF, **TNF-** **α**, EPO, TPO, TSLP, SCF, GH, **Prolactin**,** Insulin**, **LPS**, **CpG DNA**

SOCS-2	IL-2, IL-6, EPO, GH, Prolactin, Insulin, CNTF,	IL-6, **GH**,** IGF-I**

SOCS-3	IL-1, IL-2, IL-3, IL-4, IL-6, IL-9, IL-10, IL-11, IL-12, IL-22, IL-23, IL-27, IFN-*α*/*β*, IFN-*γ*, LIF, TNF-*α*, TGF-*β*, EPO, TPO, G-CSF, GH, TSH, Prolactin, Leptin, CNTF, Cardiotrophin, EGF, Insulin	IL-1, IL-2, IL-4, **IL-6**, IL-9, IL-11, **IL-27**, IFN-*α*/*β*, IFN-*γ*, **LIF**, OSM, EPO, **G-CSF**, GH, Prolactin, **Leptin**, CNTF, Cardiotrophin, **Insulin**, LPS

SOCS-4	EGF	EGF

SOCS-5	EGF	IL-4, IL-6, EGF

SOCS-6	SCF, Insulin	SCF, Insulin

SOCS-7	Insulin, IGF-1	**Insulin**

*Signaling shown in red is demonstrated in the studies using knockout mice.

**Table 2:** Proposed involvement of SOCS1 in liver pathophysiology.

	Mechanism for SOCS1 dysregulation	Pathogenic consequences in liver
Increased SOCS1	(1) Excessive stimulation with cytokines/TLR ligands	Insulin resistance
		Reduced liver regeneration

Reduced SOCS1	(1) CpG methylation	Hepatitis
	(2) Promoter polymorphism?	Liver fibrosis
	(3) Genetic mutation?	Hepatocellular carcinoma
